# Supplementary material for: Surface Microbiome Profiling of Dental Elevators Using Third‐Generation Sequencing: Implications for Infection Control in Dental Practice
Source: Microbiologyopen. 2025 Dec 8;14(6):e70178. doi: 10.1002/mbo3.70178 (PMC12685768; doi:10.1002/mbo3.70178)
Supplement: Supplementary file 1 — customer_backup. [file MBO3-14-e70178-s001.zip › customer_backup/customer_backup/function_analysis/KEGG/allsample/K2species/allsample.reabundance_Class2Genus.txt.pdf]

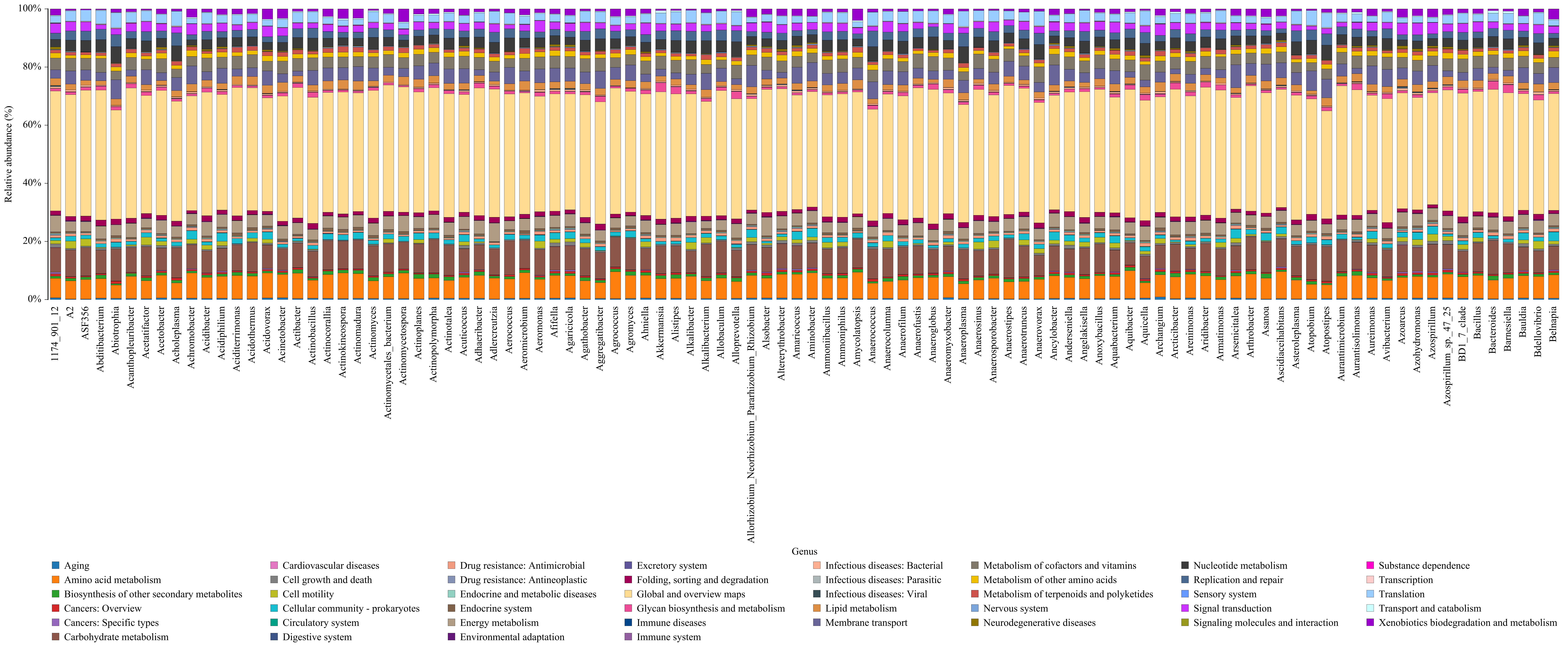
Relative abundance (%)
100%
80%
60%
40%
20%
0%
1174_901_12
A2
ASF356
Abditibacterium
Abiotrophia
Acanthopleuribacter
Acetatifactor
Acetobacter
Acholeplasma
Achromobacter
Acidibacter
Acidiphilium
Aciditerrimonas
Acidothermus
Acidovorax
Acinetobacter
Actibacter
Actinobacillus
Actinocorallia
Actinokineospora
Actinomadura
Actinomyces
Actinomycetales_bacterium
Actinomycetospora
Actinoplanes
Actinopolymorpha
Actinotalea
Acuticoccus
Adhaeribacter
Adlercreutzia
Aerococcus
Aeromicrobium
Aeromonas
Afifella
Agaricicola
Agathobacter
Aggregatibacter
Agrococcus
Agromyces
Ahniella
Akkermansia
Alistipes
Alkalibacter
Alkalibacterium
Allobaculum
Alloprevotella
Allorhizobium_Neorhizobium_Pararhizobium_Rhizobium
Alsobacter
Altererythrobacter
Amaricoccus
Aminobacter
Ammoniibacillus
Ammoniphilus
Amycolatopsis
Anaerococcus
Anaerocolumna
Anaerofilum
Anaerofustis
Anaeroglobus
Anaeromyxobacter
Anaeroplasma
Anaerosinus
Anaerosporobacter
Anaerostipes
Anaerotruncus
Anaerovorax
Ancylobacter
Anderseniella
Angelakisella
Anoxybacillus
Aquabacterium
Aquibacter
Aquicella
Archangium
Arcticibacter
Arenimonas
Aridibacter
Armatimonas
Arsenicitalea
Arthrobacter
Asanoa
Ascidiaceihabitans
Asteroleplasma
Atopobium
Atopostipes
Aurantimicrobium
Aurantisolimonas
Aureimonas
Avibacterium
Azoarcus
Azohydromonas
Azospirillum
Azospirillum_sp._47_25
BD1_7_clade
Bacillus
Bacteroides
Barnesiella
Bauldia
Bdellovibrio
Belnapia
Genus
Aging
Amino acid metabolism
Biosynthesis of other secondary metabolites
Cancers: Overview
Cancers: Specific types
Carbohydrate metabolism
Cardiovascular diseases
Cell growth and death
Cell motility
Cellular community - prokaryotes
Circulatory system
Digestive system
Drug resistance: Antimicrobial
Drug resistance: Antineoplastic
Endocrine and metabolic diseases
Endocrine system
Energy metabolism
Environmental adaptation
Excretory system
Folding, sorting and degradation
Global and overview maps
Glycan biosynthesis and metabolism
Immune diseases
Immune system
Infectious diseases: Bacterial
Infectious diseases: Parasitic
Infectious diseases: Viral
Lipid metabolism
Membrane transport
Metabolism of cofactors and vitamins
Metabolism of other amino acids
Metabolism of terpenoids and polyketides
Nervous system
Neurodegenerative diseases
Nucleotide metabolism
Replication and repair
Sensory system
Signal transduction
Signaling molecules and interaction
Substance dependence
Transcription
Translation
Transport and catabolism
Xenobiotics biodegradation and metabolism
